# Effects of *Nigella Sativa* on Type-2 Diabetes Mellitus: A Systematic Review

**DOI:** 10.3390/ijerph16244911

**Published:** 2019-12-05

**Authors:** Amiza Hamdan, Ruszymah Haji Idrus, Mohd Helmy Mokhtar

**Affiliations:** Department of Physiology, Faculty of Medicine, Universiti Kebangsaan Malaysia, Kuala Lumpur 56000, Malaysia; amizahamdan@gmail.com (A.H.); ruszyidrus@gmail.com (R.H.I.)

**Keywords:** *Nigella sativa*, Type-2 diabetes mellitus, thymoquinone, antidiabetic, hypoglycemic effect

## Abstract

Diabetes mellitus is one of the most prevalent metabolic disorders that affect people of all genders, ages, and races. Medicinal herbs have gained wide attention from researchers and have been considered to be a beneficial adjuvant agent to oral antidiabetic drugs because of their integrated effects. Concerning the various beneficial effects of *Nigella sativa*, this systematic review aims to provide comprehensive information on the effects of *Nigella sativa* on glucose and insulin profile status in humans. A computerized database search performed through Scopus and Medline via Ebscohost with the following set of keywords: *Nigella Sativa* OR black seed oil OR thymoquinone OR black cumin AND diabetes mellitus OR hyperglycemia OR blood glucose OR hemoglobin A1C had returned 875 relevant articles. A total of seven articles were retrieved for further assessment and underwent data extraction to be included in this review. *Nigella sativa* was shown to significantly improve laboratory parameters of hyperglycemia and diabetes control after treatment with a significant fall in fasting blood glucose, blood glucose level 2 h postprandial, glycated hemoglobin, and insulin resistance, and a rise in serum insulin. In conclusion, these findings suggested that *Nigella sativa* could be used as an adjuvant for oral antidiabetic drugs in diabetes control.

## 1. Introduction

Diabetes mellitus (DM) is one of the most prevalent metabolic disorders that occur throughout the world, which affects people of all genders, ages, and races. Type-2 diabetes mellitus (T2DM) accounts for 90% of all DM cases as a result of the interaction between genetic predisposition and environmental factors [1]. T2DM is characterized by the defect in insulin secretion, an action that causes an elevation in blood glucose levels. This elevation promotes the production of reactive oxygen species (ROS), which cause cellular damage that promote the development of diabetic complications, including diabetic retinopathy, nephropathy, and autonomic neuropathy [2,3].

Besides that, T2DM can also be associated with cardiovascular risk factors such as dyslipidemia, hypertension, and obesity. Measurements of fasting blood glucose (FBG), blood glucose level 2 h postprandial (2hPG), and glycated hemoglobin (HbA1c) have been used to assess glycemic control in patients with T2DM [4]. Medicinal herbs have gained wide attention from researchers and have been considered to be a beneficial adjuvant agent to oral antidiabetic drugs because of their integrated effects [5,6]. Besides that, *Nigella sativa* (NS) has also been considered to be safer compared to oral antidiabetic drugs [7]. NS is an annual herbaceous flowering plant, belonging to the Rununculaceae family which can be found mostly in Middle Eastern countries. It is also known as “black seed” or “kalonji” and has been widely used in food as spices and condiments. Different forms of NS like extract, oil, and powder have been utilized in traditional medicine to treat several illnesses such as fever, cough, diarrhea, bronchitis, and gastrointestinal diseases [8,9]. NS was reported to possess various therapeutic effects such as antidiabetic, antioxidant, anticancer, hypolipidemic, and anti-inflammatory properties [10]. 

Screening for novel bioactive compounds from NS has gained researchers’ attention due to its therapeutic effects. The therapeutic effects of NS are mainly contributed to thymoquinone, which is one of the major bioactive compounds that was discovered to have a protective effect against diabetes [11]. Previous studies revealed that thymoquinone induced a marked decrease in FBG and a marked increase in insulin levels in rats [12]. The other compounds, namely thymol, thymohydroquinone, dithymoquinone, nigellone, alpha-hederin, flavonoids, and fatty acids were also found to have participated in the therapeutic properties of NS [13]. The efficacy of NS therapeutic properties is contributed by the synergistic effect between the different compounds presents in the plant extracts. Furthermore, NS was shown to have no severe side effects or toxicological effects in human and animal models [14].

Concerning the various beneficial effects of NS, this systematic review was performed to provide comprehensive information on the effect of supplementation of NS in combination with oral antidiabetic drugs on glucose and insulin profile status in humans. Besides that, it may help to establish NS as a beneficial adjuvant to oral antidiabetic drugs for diabetes patients. 

## 2. Materials and Methods 

### 2.1. Literature Review

A systematic review of the literature was conducted to identify relevant studies about the reported effect of *Nigella sativa* (NS) on Type-2 diabetes mellitus (T2DM). To conduct a comprehensive search of biomedical science journals, two databases, namely Scopus (published between 1823 and November 2019) and Medline via Ebscohost (published between 1865 and November 2019) were used. The search strategy involved a combination of the following two sets of keywords *Nigella sativa* OR black seed oil OR thymoquinone OR black cumin AND diabetes mellitus OR hyperglycemia OR blood glucose OR hemoglobin A1C

### 2.2. Selection of Research Articles

The results were limited to original articles that were published in English and included abstracts. Review articles, news, case reports, and other original articles not associated with NS and T2DM were excluded from the review. For this review, only studies that reported the association of NS and T2DM were included.

### 2.3. Inclusion and Exclusion Criteria

For this review, only studies that reported the direct effect of NS on T2DM was included. The inclusion criteria were as follows: (i) Clinical human studies including but not limited to observational study, randomized controlled trial, AND double-blind randomized controlled trial or single-blind, non-randomized controlled trial design, AND (ii) studies involving human adult subjects, AND (iii) studies examining the effects of a crude plant preparation and not its bioactive components, AND (iv) research that reported sufficient information on at least one T2DM blood parameter, such as fasting blood glucose, HbA1c or blood glucose level 2 h postprandial (2hPG), AND (v) studies that examined the antidiabetic effects of any form of NS (powder, oil, capsule, or tea).

The following exclusions were also considered: (i) studies that investigated the impacts of an individual bioactive component of NS, OR (ii) studies on NS in combination with other herbs of ingredients as a mixture, OR (iii) studies on animals, OR (iv) studies on Type-1 diabetes mellitus (T1DM) or gestational diabetes (GDM).

### 2.4. Data Extraction and Management

Papers were screened in three phases before they were included in the review. In the first phase, studies that did not match the inclusion criteria based solely on the title were excluded. In the second phase, abstracts of the remaining studies were screened, and studies that did not meet the inclusion criteria were excluded. In order to standardize the data collection, all data extraction was performed independently with the use of a data extraction form. The following data were recorded from the studies: Type of NS used, treatment groups, biochemical parameters measured, a summary of T2DM related outcome measures, and a conclusion of the study.

## 3. Results

### 3.1. Search Results

**Figure 1:**
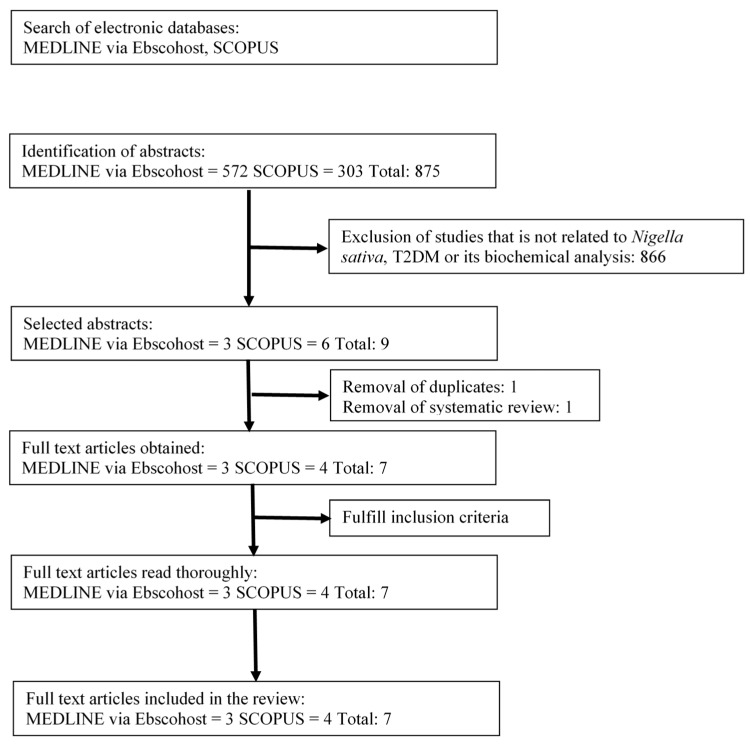
Flowchart of the selection process.

The literature searches identified 875 potentially relevant articles. Of those, 866 articles were excluded because they were not associated with *Nigella sativa* (NS) or its bioactive compound and were not related to T2DM. The search was limited to original articles written in the English, with an abstract available, and studies only in humans with T2DM and combined treatment of an oral antidiabetic drug with NS. From the remaining nine articles, one duplicate article and one systematic review article was removed before the full papers were retrieved for thorough reading. A total of seven articles were retrieved for further assessment and underwent data extraction to be included in this review. A flow chart of the selection process, including reasons for exclusion, is shown in Figure 1.

### 3.2. Study Characteristics

The summary of the characteristics of all studies is displayed in Table 1. All studies were published between the year 2009 and 2015. Four different types of NS form were used for the treatment which include oil (*n* = 3) [15,16,17], capsule (*n* = 2) [18,19], powder (*n* = 1) [20], and tea (*n* = 1) [21]. Different treatments of NS were used in the studies with the treatment of the oil form including varying doses of 0.7 g/day, 3 g/day, and 5 mL/day. For capsule form, the treatments were in the range of 1 to 3 g/day, while the treatments for powder form and tea forms were 2 g/day and 5 g/day, respectively.

In this study, we reviewed the analysis of blood samples such as fasting blood glucose (FBG), blood glucose level 2 h postprandial (2hPG), glycated hemoglobin (HbA1c), as well as insulin level and insulin resistance.

### 3.3. Fasting Blood Glucose (FBG)

From the seven studies reviewed, six studies measured the changes of the fasting blood glucose (FBG) parameter. According to Ahmad et al. (2009), FBG levels significantly decreased after treatment with NS oil for 40 days [17]. Bamosa et al. (2010) showed a significant reduction in FBG after 12 weeks of treatment with 2 g/day NS [19]. El-Shamy et al. (2011) reported a highly significant reduction of FBG in patients receiving 5 gm/day of NS tea for 6 months [21]. Hosseini et al. (2013) recorded a significant FBG decrease in the group receiving 2.5 mL NS oil [16]. Kaatabi et al. (2015) also recorded a highly significant decrease in FBG after 12 months of treatment with NS capsule 2 g/day, and Heshmati et al. (2015) showed a significant decrease in FBG levels after treatment with NS oil in soft gel capsule 3 g/day for 12 weeks [15,18].

### 3.4. Blood Glucose Level 2 h Postprandial (2hPG)

Of the seven studies reviewed, three articles studied the blood glucose level 2 h postprandial (2hPG) parameter. Bamosa et al. (2010) reported a significant decrease in 2hPG after 4 and 8 weeks of treatment with 2 g/day NS [19]. El-Shamy et al. (2011) also recorded a decrease in 2hPG levels after consumption of 5 gm/day NS tea for 6 months [21]. Meanwhile, Hosseini et al. (2013) revealed a significant decrease in 2hPG levels in the group receiving 2.5 mL NS oil compared with the placebo group [16].

### 3.5. Glycated Hemoglobin (HbA1c)

Of the seven papers reviewed, six articles investigated HbA1c. Bamosa et al. (2010) reported a significant decrease in HbA1c levels following 12 weeks treatment with 2 g/day and 3 g/day of NS [19]. El Shamy et al. (2011) showed a significant decrease of HbA1c levels after consumption of 5 gm/day NS tea for 6 months [21]. Hosseini MS et al. (2013) also reported NS oil at dose 5 mL/day significantly decreased HbA1c levels, which is in line with Kaatabi et al. (2015) who reported treatment with NS capsule 2 g/day significantly decreased HbA1c levels after 12 months treatment [16,18]. Heshmati et al. (2015) and Bamosa et al. (2015) also showed similar findings with the treatment of NS significantly reducing the HbA1c levels [15,20].

### 3.6. Insulin Level and Insulin Resistance

Of the seven papers reviewed, four articles studied insulin level and insulin resistance. Ahmad et al. (2009) showed that the insulin levels significantly increased after treatment with NS oil for 40 d [17]. NS at dose 2 g/day significantly decreased insulin resistance index and increased *β*-cell function after 12 weeks of treatment [19]. According to Kaatabi et al. (2015), 2 g/day NS capsule significantly decreased insulin resistance after 3, 6, 9, and 12 months treatment [18]. Meanwhile, Hesmati et al. (2015) revealed that insulin levels and insulin resistance decreased in the intervention group, but they were not significant after adjusting for confounder factors [15].

## 4. Discussion

Plants have long been used as medicinal herbs in Arabian countries, Asia, and Africa [22]. To date, there has been growing attention on the use of medicinal herbs as an alternative to the standard care in treating diseases, mainly diabetes. Extensive investigations have been conducted to evaluate the potential of medicinal herbs in treating diseases. Only 15% of the total of 300,000 medicinal herbs have been explored for their pharmacological properties [23]. Among the various naturally medicinal herbs, *Nigella sativa* (NS) has a promising future in the prevention and treatment of diabetic diseases due to its capacity to treat multiple diseases, as well as being cost-efficient and having fewer side effects compared to synthetic medicines [24]. 

In this systematic review, we have assessed the effects of NS supplementation on fasting blood glucose (FBG), blood glucose level 2 h postprandial (2hPG), glycated hemoglobin (HbA1c), insulin level, and insulin resistance. NS was given as an adjuvant therapy in patients with Type-2 diabetes mellitus (T2DM) in addition to their oral antidiabetic drug. 

The literature search revealed six studies that showed treatment with different forms of NS caused marked reduction in the level of FBG and HbA1c compared to the control group [15,16,17,18,19,21]. Meanwhile, three studies reported NS treatments significantly reduced 2hPG compared to the corresponding baseline [16,19,21]. These findings reflect the potential use of NS as a complementary treatment for T2DM. This assumption is further confirmed by studies conducted using animal models, in which treatment of NS for 1 month significantly reduced FBG in streptozotocin-induced diabetic rats [25]. Reduction in HbA1c levels could also be seen in streptozotocin-nicotinamide induced diabetic rats treated with thymoquinone [26]. Najmi et al. (2008) reported that treatment of 2.5 mL NS oil twice daily for 6 weeks had shown an improvement on FBG, suggesting that NS oil might be a beneficial adjuvant to the oral antidiabetic drug [27]. Besides that, treatment of 1.5 and 3 mL/day NS oil for 20 d resulted in a reduction of HbA1c and FBG levels in patients with metabolic syndrome risks [28]. HbA1c is an indicator of long-term glycemic control due to its ability to reflect the cumulative glycemic history of the preceding 2 to 3 months. It also correlates with the risk of long-term diabetes complications. Thus, a reduction in HbA1c could reduce the risk of diabetes complications [29]. 

It has been reported that chronic elevation of blood glucose promotes oxidative stress through overproduction of reactive oxygen species (ROS). Excessive levels of ROS cause an increase in insulin resistance and β-cell dysfunction, thus contributing to the advancement of diabetic complications [30,31,32]. Pancreatic β-cells are easily destructed by oxidative stress due to the low level of free-radical quenching enzymes, which will decrease insulin secretion [33].

Most of the studies reviewed demonstrated NS supplementation combined with oral antidiabetic drugs enhanced glycemic control manifested by improvement in pancreatic β-cell function and insulin resistance compared to the corresponding baseline. Based on findings by Bamosa et al. (2010), the insulin resistance was significantly reduced by 2 g daily supplementation of NS [19]. The same treatment of NS also produced a significant increase in β-cell function. These findings were in line with the study by Nehar and Kumari (2013), which showed the NS seed extract can ameliorate insulin resistance, thus reducing blood glucose levels [34]. Besides that, Fararh et al. (2004) also highlighted the treatment of NS oil for 4 weeks in streptozotocin plus nicotinamide-induced diabetic hamsters caused a significant increase in serum insulin levels [35]. These hypoglycemic effects of NS have been reported to be regulated through the activation of insulin and AMP-activated protein kinase (AMPK) pathways [36]. 

Hypoglycemic effects of NS have been attributed to its antioxidant properties [37]. Antioxidants are one of the potential strategies for diabetic treatment. Thymoquinone is a major antioxidant component of NS which possesses antioxidant potential that can scavenge free radicals [38]. It could reduce oxidative stress and promote the proliferation of pancreatic β-cell integrity, thus leading to the improvement in insulin secretion [39]. Besides that, hypoglycemic effects of thymoquinone contribute by its downregulating effect on the gluconeogenic enzymes expression and its ability in reducing intestinal absorption for glucose [40]. It may also inhibit gluconeogenesis via activating adenosine monophosphate-activated protein kinase (AMPK) in muscles and the liver [41].

This systematic review also revealed different that forms of *Nigella sativa* such as oil, water extract, and powder produce similar hypoglycemic effects, in which these treatments resulted in significantly reduced HbA1c levels. This finding suggests that other constituents in *Nigella sativa* seed also possess the same beneficial properties as thymoquinone which is a major component of NS oil fraction [42].

Some limitations are notable in this systematic review and must be considered. This current study included a small number of relevant studies. Besides that, all included studies utilized different preparations, doses, and duration of supplementation. Thus, our findings on the efficacies of supplementation of NS in combination with oral antidiabetic drugs should be interpreted with caution.

## 5. Conclusions

All studies included in this review reported positive effect of NS on T2DM. NS was showed to improve laboratory parameters of hyperglycemia and diabetes control with a significant fall in fasting blood glucose, and a significant rise in serum insulin. These findings suggest that NS could be used as an adjuvant for oral antidiabetic drugs in diabetes control. However, further studies are needed since it is difficult to determine effective type and dosage of NS in diabetes management due to chemical compositions of different sources of NS, dosage, and duration of intervention. 

## Figures and Tables

**Table 1:** Effects of *Nigella sativa* on Type-2 diabetes mellitus.

REF	Study Design	Type of *Nigella sativa*	Methodology		T2DM Related Biochemical Outcome	Conclusions
			Treatment Group	Biochemical Analysis		
[17]	Interventional study (pre-post study).	*Nigella sativa* oil (from 0.7 g *Nigella sativa* seeds purchased from Rawalpindi, Pakistan) to be consumed orally for 40 d.	Forty-one T2DM patients consumed *Nigella sativa* oil for the 1st 40 d (NS treatment) followed by wheat bran for the 2nd 40 d (placebo), combined with their usual oral antidiabetic drug at constant dose.	Analysis of blood samples: Fasting blood glucose (FBG), insulin level blood urea, platelet count, total leucocytes count (TLC), serum alanine aminotransferase (ALT), and serum aspartate aminotransferase (AST).	FBG significantly decreased from 190.780 ± 8.042 mg/dL to 168.317 ± 7.150 mg/dL following the 1st treatment before significantly increasing back to 186.487 ± 7.491 mg/dL after the 2nd treatment. Although not significant, insulin levels increased from 8.013 ± 0.758 lU/mL to 13.194 ± 1.404 ulU/mL after the 1st treatment before decreasing back to 8.850 ± 0.694 ulU/mL after the 2nd treatment.	*Nigella sativa* oil decreased FBG and increased insulin levels when combined with oral antidiabetic drug.
[19]	Interventional study (pre-post study).	*Nigella sativa* seed (Bioextract (Pvt) Ltd, Sri Lanka) in capsules contain 500 mg of grounded *Nigella sativa* to be consumed orally in three different doses 1, 2, and 3 g/day for 12 weeks.	Ninety-four T2DM patients divided randomly into 3 groups. Thirty patients receiving 1 g/day dose, 32 patients receiving 2 g/day dose, and 32 patients receiving 3 g/day dose together with their oral antidiabetic drug for 4, 8, and 12 weeks.	Analysis of blood samples: FBG, blood glucose level 2 h postprandial (2hPG), glycated hemoglobin (HbA1c), insulin resistance index, β-cell function, serum C-peptide, and body mass index (BMI).	(a) FBG at 0, 4, 8, and 12 weeks showed no significant changes with the 1 g/day capsule with 189 ± 14.3, 186 ± 38, 171 ± 10.1, and 171 ± 7.8 mg/dL, respectively. With 2 g/day capsule, FBG at 0, 4, 8, and 12 weeks showed a significant reduction from 219 ± 12.3, 174 ± 10.1, 157 ± 10.8, to 162 ± 9.2 mg/dL accordingly. With 3 g/day capsule, FBG at 0, 4, 8, and 12 weeks showed a significant reduction from 204 ± 18.2, 176 ± 15.2, 157 ± 9.9, to 169 ± 16.4 mg/dL, respectively. (b) 2hPG levels at 0, 4, 8, and 12 weeks did not change with 1 g/day capsule with readings of 286 ± 23.3, 244 ± 22.5, 241 ± 19.2, and 218 ± 15.6 mg/dL, respectively. With 2 g/day capsule, 2hPG levels at 0, 4, 8, and 12 weeks decreased significantly with readings of 289 ± 24.2, 213 ± 27.8, 231 ± 26.58, and 256 ± 28.1 mg/dL, respectively. Although not significant, 2hPG levels increased with 3 g/day capsule with readings at 0, 4, 8, and 12 weeks of 277 ± 54.3, 301 ± 54.3, 229 ± 9.9, and 234 ± 80.3 mg/dL, respectively. (c) HbA1c at 12 weeks did not significantly decrease from baseline with 1 g/day capsule, with readings from 8.36 ± 0.31 to 8.01 ± 0.27 %. With 2 g/day capsule, HbA1c decreased significantly from 9.09 ± 0.24 to 7.57 ± 0.30 %. With 3 g/day capsule, HbA1c decreased significantly from 9.35 ± 0.41 to 7.31 ± 0.37 %. (d) Insulin resistance index did not significantly change from 2.75 ± 0.34 to 2.82 ± 0.26 with 1 g/day capsule. With 2 g/day capsule, insulin resistance index significantly decreased at 12 weeks from 3.20 ± 0.36 to 2.37 ± 0.20. Although not significant, with 3 g/day capsule, insulin resistance index increased at 12 weeks from 4.11 ± 0.55 to 2.98 ± 0.49. (e) β-cell function decreased with 1 g/day capsule from 61.75 ± 7.79 to 59.12 ± 8.19, while it increased with 2 g/day and 3 g/day capsule, going from 45.03 ± 6.28 to 63.63 ± 9.59 and from 41.89 ± 9.83 to 88.90 ± 36.05, respectively. All changes in β-cell function were not statistically significant.	*Nigella sativa* at dose 2 g/day significantly improves diabetic control when combined with oral antidiabetic drug.
[21]	Interventional study (pre-post study).	*Nigella sativa* tea. Extract by hot water. The tea was 5 g/day for 6 months.	Sixty-six T2DM patients divided into 2 groups. Forty-one T2DM patients (diabetic group) and 25 healthy peoples (normal group). T2DM patients combined their oral antidiabetic drug with *Nigella sativa* tea.	Analysis of blood samples: FBG; 2hPG; HbA1c, kidney function test (serum creatinine), kidney function test (blood urea), liver function test (AST), liver function test (ALT), serum total bilirubin, direct bilirubin, and indirect bilirubin.	(a) FBG for the normal group: FBG significantly decreased from 80.22 ± 10.8 to 78.14 ± 10.3 after 1 month, highly significant decrease to 76.79 ± 8.66 after 2 months, very highly significant decrease to 75.30 ± 8.97 after 3 months and after 6 months to 73.34 ± 8.71. FBG for the diabetic group: Very highly significant decrease in FBG for all months from 148.7 ± 26.59 to 137.93 ± 28.36 after 1 month, to 131.64 ± 26.33 after 2 months, to 126.46 ± 23.14 after 3 months, and to 127.67 ± 22.01 after 6 months. *Nigella sativa* tea significantly decreased FBG for the diabetic group after 3 months, and also 6 months for normal group. (b) 2hPG for the normal group: Very highly significant decrease in 2hPG for all months from 101.13 ± 15.25 to 96.01 ± 14.12 after 1 month, to 93.16 ± 12.93 after 2 months, to 92.20 ± 13.58 after 3 months, and 89.49 ± 12.38 after 6 months. 2hPG for the diabetic group: Very highly significant decrease in 2hPG for all months from 251.42 ± 76.88 to 216.39±61.09 after 1 months, to 192.86 ± 46.11 after 2 months, to 174.27 ± 36.60 after 3 months, and to 164.12 ± 28.72 after 6 months. *Nigella sativa* tea very highly significant decrease in 2hPG for normal and diabetic groups. (c) HbA1c for the normal group: No significant decrease in HbA1c from 4.43±0.36 to 4.26 ± 0.51 after 3 months, highly significant decrease to 4.14 ± 0.47 after 6 months. HbA1c for the diabetic group: Very highly significant decrease in HbA1c from 7.18 ± 0.83 to 6.59 ± 0.62 after 3 months and to 6.02 ± 0.58 after 6 months. *Nigella sativa* tea very highly significant decrease in HbA1c after 3 and 6 months treatment.	*Nigella sativa* tea extract 5 g/day showed improvement in blood glucose levels and when combined with oral antidiabetic drug.
[16]	Randomized, double-blind, and controlled trial.	*Nigella sativa* oil purchased from local market in Tehran city. Cold press procedure is used to produce the *Nigella sativa* oil. 5 mL *Nigella sativa* per day for 3 months.	Seventy T2DM patients divided into 2 groups of 35 each. Group 1 (*Nigella sativa* group - *Nigella sativa* oil), group 2 (placebo group-mineral oil). All subjects taken oral antidiabetic drug combined with *Nigella sativa* oil.	Analysis of blood samples: FBG, 2hPG, HbA1C, creatinine, cholesterol, triglyceride, high-density lipoproteins (HDL); low-density lipoproteins (LDL), body mass index (BMI), serum glutamic oxaloacetic transaminase (SGOT), serum glutamic pyruvic transaminase (SGPT), and alkaline phosphatase (ALP).	(a) FBG for group 1: Significant decrease in FBG from 180.2 ± 31.8 to 161.9 ± 45.3. FBG for group 2: Increased FBG from 179.8 ± 32.3 to 186.3 ± 42.1. *Nigella sativa* oil significantly decreased FBG after treatment combined with oral antidiabetic drug. (b) 2hPG for group 1: Significant decrease in 2hPG from 183.0 ± 38.7 to 167.9 ± 37.5. 2hPG for group 2: Increase in 2hPG from 189.7 ± 42.8 to 192.2 ± 41.7. *Nigella sativa* oil significantly decreased 2hPG for Group 1. (c) HbA1c for group 1: Significant decrease in HbA1c from 8.82 ± 0.73 to 8.52 ± 0.68. HbA1c for group 2: Decrease in HbA1c from 8.79 ± 0.55 to 8.70 ± 0.67. *Nigella sativa* oil significantly decreased HbA1c for Group 1.	*Nigella sativa* oil at dose 5 mL significantly decreased FBG levels, 2hPG, and HbA1c when combined with oral antidiabetic drug.
[18]	Randomized, single-blind, and controlled trial.	*Nigella sativa* seeds as powder in capsules of 500 mg (Sri Lanka). Dose used in this study was 2 g/day.	One-hundred-and-fourteen T2DM patients divided into 2 groups. Fifty-seven patients in control group (Charcoal capsule) and 57 patients in *Nigella sativa* group. Results were taken every 3 months until 1 year. All patients continued their own oral antidiabetic drug.	Analysis of blood samples: FBG, HbA1c, insulin resistance (IR), β-cell activity, C-peptide, total antioxidant capacity (TAC), superoxide dismutase (SOD), catalase (CAT), and glutathione and thiobarbituric acid reactive substances (TBARS).	(a) FBG for the *Nigella sativa* group: Significant decrease in FBG from 195 ± 6.57 to 163 ± 6.31 after 3 months, to 164 ± 5.97 after 6 months, to 176 ± 6.59 after 9 months, and to 172 ± 5.83 after 12 months. FBG for the control group: Increase in FBG from 180 ± 5.75 to 184 ± 5.81 after 3 months, to 185 ± 5.59 after 6 months, to 183 ± 5.41 after 9 months, and to 180 ± 5.59 after 12 months. *Nigella sativa* capsule significant decrease FBG after 3, 6, 9, and 12 months for the *Nigella sativa* group. (b) HbA1c for the *Nigella sativa* group: Significant decrease in HbA1c from 8.6 ± 0.13 to 7.9 ± 0.18 after 3 months, to 7.8 ± 0.22 after 6 months, to 7.9 ± 0.19 after 9 months, and to 8.2 ± 0.14 after 12 months. HbA1c for the control group: Increase in HbA1c from 8.2 ± 0.12 to 8.3 ± 0.12 after 3 months, to 8.3 ± 0.13 after 6 months, to 8.5 ± 0.15 after 9 months, and to 8.5 ± 0.14 after 12 months. *Nigella sativa* capsule significantly decreased HbA1c after 3, 6, 9, and 12 months for the *Nigella sativa* group. (c) Insulin resistance (IR) for the *Nigella sativa* group: Significant decrease in insulin resistance from 3.0±0.24 to 2.5±0.16 after 3 months, to 2.4 ± 0.17 after 6 months, to 2.5 ± 0.19 after 9 months, and to 2.5 ± 0.18 after 12 months. IR for the control group: Increase in insulin resistance from 2.5 ± 0.17 to 2.6 ± 0.16 after 3 months, to 2.7 ± 0.19 after 6 months, to 2.7 ± 0.16 after 9 months, and to 2.5 after 12 months. *Nigella sativa* capsule significantly decreased insulin resistance after 3, 6, 9, and 12 months for the *Nigella sativa* group.	*Nigella sativa* capsule 2 g/day significantly decreased FBG, HbA1c, insulin resistance (IR) when combined with oral antidiabetic drug.
[20]	Randomized, single-blind, and controlled trial.	*Nigella sativa* powdered 2 g/day for 1 year.	Sixty T2DM patients divided into a control group that received charcoal and test group that received *Nigella sativa* powdered. Combined with oral antidiabetic drug.	Analysis of blood samples: HbA1c, BMI, pulse rate, and mean arterial pressure (MAP).	(a) HbA1c for the test group: Significant decrease in HbA1c from 8.84 ± 0.96 to 8.40 ± 1.07 after 12 months. Decrease in HbA1c from 8.78 ± 0.95 to 8.14 ± 1.69 after 6 months. HbA1c for the control group: Increase in HbA1c from 8.14 ± 0.79 to 8.28 ± 0.80 after 6 months. Increase in HbA1c from 8.18 ± 0.77 to 8.26 ± 0.90 after 12 months.	*Nigella sativa* powdered 2 g/day for 1 year significantly decreased HbA1c when combined with oral antidiabetic drug.
[15]	Randomized, double-blind, and controlled trial.	*Nigella sativa* oil in soft gel capsule 3 g/day for 12 weeks.	Seventy-two T2DM patients. Thirty-six participants in the intervention group received *Nigella sativa* oil, and 36 participants in control group received sunflower soft gel capsules. Combined with oral antidiabetic drug.	Analysis of blood samples: FBG, HbA1c, insulin, Homeostatic Model Assessment of Insulin Resistance (HOMA-IR), triglyceride, total cholesterol, HDL-cholesterol, and LDL-cholesterol.	(a) FBG for the intervention group: Significant decrease in FBG from 183.4 ± 42.1 to 166.3 ± 38.5. FBG for the control group: Increase in FBG from 201.8 ± 63.9 to 204.9 ± 63.2. *Nigella sativa* soft gel capsule significantly decreased FBG. (b) HbA1c for the intervention group: Significant decrease in HbA1c from 8.3 ± 0.9 to 7.8 ± 0.8. HbA1c for the control group: Increase in HbA1c from 8.3 ± 1.0 to 8.6 ± 1.0 *Nigella sativa* soft gel capsule significantly decreased HbA1c.	*Nigella sativa* oil in soft gel capsule 3 g/day significantly decreased FBG and HbA1c when combined with oral antidiabetic drug.

## References

[1] Reimann M., Bonifacio E., Solimena M., Schwarz P.E., Ludwig B., Hanefeld M., Bornstein S.R. (2009). An update on preventive and regenerative therapies in diabetes mellitus. Pharmacol. Ther..

[2] Fiorentino T.V., Prioletta A., Zuo P., Folli F. (2013). Hyperglycemia-induced oxidative stress and its role in diabetes mellitus related cardiovascular diseases. Curr. Pharm. Des..

[3] Aronson D. (2008). Hyperglycemia and the pathobiology of diabetic complications. Adv. Cardiol..

[4] Monnier L., Colette C., Dunseath G.J., Owens D.R. (2007). The loss of postprandial glycemic control precedes stepwise deterioration of fasting with worsening diabetes. Diabetes Care.

[5] Derosa G., Putignano P., Bossi A.C., Bonaventura A., Querci F., Franzetti I.G., Guazzini B., Testori G., Fogari E., Maffioli P. (2011). Exenatide or glimepiride added to metformin on metabolic control and on insulin resistance in type 2 diabetic patients. Eur. J. Pharmacol..

[6] Pan S.-Y., Zhou S.-F., Gao S.-H., Yu Z.-L., Zhang S.-F., Tang M.-K., Sun J.-N., Ma D.-L., Han Y.-F., Fong W.-F. (2013). New Perspectives on How to Discover Drugs from Herbal Medicines: CAM’s Outstanding Contribution to Modern Therapeutics. Evid. Based Complement. Altern. Med..

[7] Ahmad A., Husain A., Mujeeb M., Khan S.A., Najmi A.K., Siddique N.A., Damanhouri Z.A., Anwar F. (2013). A review on therapeutic potential of *Nigella sativa*: A miracle herb. Asian Pac. J. Trop. Biomed..

[8] Ramadan M.F. (2007). Nutritional value, functional properties and nutraceutical applications of black cumin (*Nigella sativa* L.): An overview. Int. J. Food Sci. Technol..

[9] Ali B.H., Blunden G. (2003). Pharmacological and toxicological properties of *Nigella sativa*. Phytother. Res..

[10] Farkhondeh T., Samarghandian S., Borji A. (2017). An overview on cardioprotective and anti-diabetic effects of thymoquinone. Asian Pac. J. Trop. Med..

[11] Khader M., Eckl P.M. (2014). Thymoquinone: An emerging natural drug with a wide range of medical applications. Iran. J. Basic Med Sci..

[12] Abdelrazek H.M.A., Kilany O.E., Muhammad M.A.A., Tag H.M., Abdelazim A.M. (2018). Black Seed Thymoquinone Improved Insulin Secretion, Hepatic Glycogen Storage, and Oxidative Stress in Streptozotocin-Induced Diabetic Male Wistar Rats. Oxidative Med. Cell. Longev..

[13] Daryabeygi-Khotbehsara R., Golzarand M., Ghaffari M.P., Djafarian K. (2017). *Nigella sativa* improves glucose homeostasis and serum lipids in type 2 diabetes: A systematic review and meta-analysis. Complement. Ther. Med..

[14] Yimer E.M., Tuem K.B., Karim A., Ur-Rehman N., Anwar F. (2019). *Nigella sativa* L. (*Black Cumin*): A Promising Natural Remedy for Wide Range of Illnesses. Evid. Based Complement. Altern. Med..

[15] Heshmati J., Namazi N. (2015). Effects of black seed (*Nigella sativa*) on metabolic parameters in diabetes mellitus: A systematic review. Complement. Ther. Med..

[16] Hosseini M.S., Mirkarimi S.A., Amini M., Mohtashami R., Kianbakht S., Fallah Huseini H. (2013). Effects of *Nigella sativa* L. Seed Oil in Type II Diabetic Patients: A Randomized, Double-Blind, Placebo Controlled Clinical Trial. JMPIR.

[17] Ahmad B., Tariq M., Uppal A.M., Naveed A.K. (2009). Effects of *Nigella sativa* oil on some blood parameters in type 2 diabetes mellitus patients. Asian J. Chem..

[18] Kaatabi H., Bamosa A.O., Badar A., Al-Elq A., Abou-Hozaifa B., Lebda F., Al-Khadra A., Al-Almaie S. (2015). *Nigella sativa* improves glycemic control and ameliorates oxidative stress in patients with type 2 diabetes mellitus: Placebo controlled participant blinded clinical trial. PLoS ONE.

[19] Bamosa A.O., Kaatabi H., Lebdaa F.M., Elq A.M., Al-Sultanb A. (2010). Effect of *Nigella sativa* seeds on the glycemic control of patients with type 2 diabetes mellitus. Indian J. Physiol. Pharmacol..

[20] Bamosa A., Kaatabi H., Badar A., Al-Khadra A., Al Elq A., Abou-Hozaifa B., Lebda F., Al-Almaie S. (2015). *Nigella sativa*: A potential natural protective agent against cardiac dysfunction in patients with type 2 diabetes mellitus. J. Fam. Community Med..

[21] El-Shamy K.A., Mosa M.M.A., El-Nabarawy S.K., El-Qattan M. (2011). Effect of *Nigella sativa* tea in type 2-diabetic patients as regards glucose homeostasis, liver and kidney functions. J. Appl. Sci. Res..

[22] Javed S. (2012). Nutritional, phytochemical potential and pharmacological evaluation of Nigella Sativa (*Kalonji*) and Trachyspermum Ammi (*Ajwain*). J. Med. Plants Res..

[23] De Luca V., Salim V., Atsumi S.M., Yu F. (2012). Mining the Biodiversity of Plants: A Revolution in the Making. Science.

[24] Aisa H.A., Xin X., Tang D., Watson R.R., Preedy V.R. (2019). Chapter 40-*Nigella sativa*: A Medicinal and Edible Plant That Ameliorates Diabetes. Bioactive Food as Dietary Interventions for Diabetes.

[25] El Rabey H.A., Al-Seeni M.N., Bakhashwain A.S. (2017). The Antidiabetic Activity of *Nigella sativa* and Propolis on Streptozotocin-Induced Diabetes and Diabetic Nephropathy in Male Rats. Evid. Based Complement. Altern. Med..

[26] Kawada T., Akiyama T., Shimizu S., Kamiya A., Uemura K., Li M., Shirai M., Sugimachi M. (2009). Detection of endogenous acetylcholine release during brief ischemia in the rabbit ventricle: A possible trigger for ischemic preconditioning. Life Sci..

[27] Najmi A., Nasiruddin M., Khan R.A., Haque S.F. (2008). Effect of *Nigella sativa* oil on various clinical and biochemical parameters of insulin resistance syndrome. Int. J. Diabetes Dev. Ctries..

[28] Rachman P.N.R., Darmawan E.A. (2017). The efficacy of black cumin seed (*Nigella sativa*) oil and hypoglycemic drug combination to reduce HbA1c level in patients with metabolic syndrome risk. IOP Conf. Ser. Mater. Sci. Eng..

[29] Stratton I.M., Adler A.I., Neil H.A.W., Matthews D.R., Manley S.E., Cull C.A., Hadden D., Turner R.C., Holman R.R. (2000). Association of glycaemia with macrovascular and microvascular complications of type 2 diabetes (UKPDS 35): Prospective observational study. BMJ.

[30] Pitocco D., Tesauro M., Alessandro R., Ghirlanda G., Cardillo C. (2013). Oxidative stress in diabetes: Implications for vascular and other complications. Int. J. Mol. Sci..

[31] Soskic S.S., Dobutovic B.D., Sudar E.M., Obradovic M.M., Nikolic D.M., Djordjevic J.D., Radak D.J., Mikhailidis D.P., Isenovic E.R. (2011). Regulation of Inducible Nitric Oxide Synthase (iNOS) and its Potential Role in Insulin Resistance, Diabetes and Heart Failure. Open Cardiovasc. Med. J..

[32] Tiganis T. (2011). Reactive oxygen species and insulin resistance: The good, the bad and the ugly. Trends Pharmacol. Sci..

[33] Tiedge M., Lortz S., Drinkgern J., Lenzen S. (1997). Relation between antioxidant enzyme gene expression and antioxidative defense status of insulin-producing cells. Diabetes.

[34] Nehar S., Kumari M. (2013). Ameliorating Effect of *Nigella sativa* Oil in Thioacetamide-induced Liver Cirrhosis in Albino Rats. Indian J. Pharm. Educ. Res..

[35] Fararh K.M., Atoji Y., Shimizu Y., Shiina T., Nikami H., Takewaki T. (2004). Mechanisms of the hypoglycaemic and immunopotentiating effects of *Nigella sativa* L. oil in streptozotocin-induced diabetic hamsters. Res. Vet. Sci..

[36] Ali B., Louis M.C., Diane V., Yara H., Pierre H.S. (2008). Antidiabetic effects of *Nigella sativa* are mediated by activation of insulin and AMPK pathways, and by mitochondrial uncoupling. Can. J. Diabetes.

[37] Badary O.A., Taha R.A., Gamal el-Din A.M., Abdel-Wahab M.H. (2003). Thymoquinone is a potent superoxide anion scavenger. Drug Chem. Toxicol..

[38] Al Wafai R.J. (2013). *Nigella sativa* and thymoquinone suppress cyclooxygenase-2 and oxidative stress in pancreatic tissue of streptozotocin-induced diabetic rats. Pancreas.

[39] Mohmoud Saleh Mansi K. (2005). Effects of Oral Administration of Water Extract of *Nigella sativa* on Serum Concentrations of Insulin and Testosterone in Alloxan-induced Diabetic Rats. Pak. J. Biol. Sci..

[40] Meddah B., Ducroc R., El Abbes Faouzi M., Eto B., Mahraoui L., Benhaddou-Andaloussi A., Martineau L.C., Cherrah Y., Haddad P.S. (2009). *Nigella sativa* inhibits intestinal glucose absorption and improves glucose tolerance in rats. J. Ethnopharmacol..

[41] Coughlan K.A., Valentine R.J., Ruderman N.B., Saha A.K. (2014). AMPK activation: A therapeutic target for type 2 diabetes?. Diabetes Metab. Syndr. Obes. Targets Ther..

[42] Geng D., Zhang S., Lan J. (2009). Analysis on chemical components of volatile oil and determination of thymoquinone from seed of Nigella glandulifera. Zhongguo Zhong Yao Za Zhi = China J. Chin. Mater. Med..

